# Phylogeography of *Angiostrongylus cantonensis* (Nematoda: Angiostrongylidae) in southern China and some surrounding areas

**DOI:** 10.1371/journal.pntd.0005776

**Published:** 2017-08-21

**Authors:** Jian Peng, Zhang-Ping He, Shuai Zhang, Zhao-Rong Lun, Zhong-Dao Wu, Chia-Kwung Fan, Christopher L. Brown, Po-Ching Cheng, Shih-Yi Peng, Ting-Bao Yang

**Affiliations:** 1 State Key Laboratory of Biocontrol, Guangdong Provincial Key Laboratory for Improved Variety Reproduction of Aquatic Economic Animals, and Center for Parasitic Organisms, School of Life Sciences, Sun Yat-sen University, Guangzhou, P.R. China; 2 Zhongshan School of Medicine, Sun Yat-sen University, Guangzhou, P.R. China; 3 Department of Molecular Parasitology and Tropical Diseases, School of Medicine, College of Medicine, Taipei Medical University, Taipei, Taiwan; 4 School of Medicine, Tzu Chi University, Hualien, Taiwan; Queen's University Belfast, UNITED KINGDOM

## Abstract

*Angiostrongylus cantonensis* is of increasing public health importance as the main zoonotic pathogen causing eosinophilic meningitis or meningoencephalitis, which has been documented all over the world. However, there are very limited studies about its phylogeography and spread pattern. In the present study, the phylogeography of *A*. *cantonensis* in southern China (including Taiwan) and partial areas of Southeast Asia were studied based on the sequences of complete mitochondrial cytochrome b (Cytb) gene. A total of 520 individuals of *A*. *cantonensis* obtained from 13 localities were sequenced for the analyses and grouped into 42 defined haplotypes. The phylogenetic tree (NJ tree and BI tree) revealed a characteristic distribution pattern of the four main lineages, with detectable geographic structure. Genetic differentiation among populations was significant, but demographic expansion could not be detected by either neutrality tests or mismatch distribution analysis, which implied a low gene flow among the local populations in different regions where the samples were collected. Two unique lineages of the *A*. *cantonensis* population in Taiwan were detected, which suggests its multiple origin in the island. Populations in Hekou (China) and Laos showed the highest genetic diversities, which were supported by both genetic diversity indices and AMOVA. These results together infer that the area around Thailand or Hekou in Yunnan province, China are the most likely origins of *Angiostrongylus cantonensis*.

## Author summary

Since it was described in 1935, more than 2800 cases of the disease have reportedly been caused by *A*. *cantonensis* worldwide, primarily in tropical and subtropical regions. Despite a relevant body of research on pathology, diagnosis and treatment, little is known about the phylogeography of *A*. *cantonensis*. Since southern China is one of the endemic regions, we performed this experiment to reveal the distribution pattern of *A*. *cantonensis* in southern China based on mitochondrial Cytb data. Our results revealed a unique pattern probably shaped by the biological features of its hosts and geographical barriers, simultaneously reflecting a low gene flow among populations. Nevertheless, the connective consanguinity between some locations (Taiwan and Southeast Asia) provides new evidence of the impact on its dispersal as influenced by human activities, indicating the emerging need of an effective strategy to control this helminth. In addition to the corresponding investigation on its hosts, more attention to the situation in southwest China and Southeast Asia is suggested to facilitate the understanding of the phylogeography of *A*. *cantonensis*.

## Introduction

The nematode *Angiostrongylus cantonensis* (Chen, 1935) is one of 21 described species in its genus, and is the zoonotic parasite responsible for human eosinophilic meningitis or meningocephalitis [1, 2]. Originally discovered in the pulmonary arteries and hearts of rats *Rattus rattus* and *R*. *norvegicus* [1], *A*. *cantonensis* has been isolated from a number of intermediate hosts including terrestrial and aquatic snails species such as *Achatina fulica* and *Pomacea canaliculata*, from which it moves on to a number of species that serve as paratenic hosts, such as crustaceans, monitor lizards and various frogs species. The *Angiostrongylus* larvae develop to the third larval stage in snails or slugs with ingestion of infected rat-feces, and the main route of human infection of this nematode is through ingestion of raw or undercooked foods containing the third stage larvae, although human beings are not the normal definitive host [3]. Thousands of cases of human eosinophilic meningitis and meningoencephalitis caused by *A*. *cantonensis* have been reported, mainly from Southeast Asia and the Pacific. Due to the deliberate or unintentional introduction of its hosts, the epidemic areas of the angiostrongyliasis have expanded to novel countries and regions including Australia and Latin America [4, 5], in an apparent correlation with the dispersal of its intermediate host, especially *Achatina fulica* infected with *A*. *cantonensis* [6]. Globally, increasing interest in the diagnosis and treatment of angiostrongyliasis has followed its recent spread, however, diagnosis and treatments of this potentially fatal disease are difficult because of the unfamiliarity with the distinguishing biological features of this worm, and the lack of awareness of food security in some regions [7, 8].

A more comprehensive understanding of the phylogeography of *A*. *cantonensis* may provide insight into the spread of this zoonotic pathogen, as exemplified by a few recent molecular phylogeographic studies on this nematode. Four species of *Angiostrongylus* spp. including *A*. *cantonensis* were distinguished based on the sequence analysis of the mitochondrial cytochrome c oxidase subunit I (COI), among which China isolates and those sampled in Thailand formed two parallel subclades [9]. Another survey based on the same gene marker COI for *A*. *cantonensis* sampled from Japan, mainland China, Taiwan, and Thailand revealed that the current geographical distribution of *A*. *cantonensis* probably reflects multiple independent origins that are likely to have been influenced by human activities [10]. Similarly, a study by Monte [11] *et al*. on phylogenetic relationship of COI for *A*. *cantonensis* demonstrated that some haplotypes from Brazil clustered with isolates from Asia, while the rest formed distinctly divergent clades, implying multiple origins of *A*. *cantonensis* in Brazil. Besides, Alicata [12] proposed that *A*. *cantonensis* originated in East Africa and spread worldwide with its host *Achatina fulica*, while Drozdz [13] considered Southeast Asia as the original source of this parasite. Thus far, no widely-accepted explanation to the spread and distribution of *A*. *cantonensis* has emerged. Nevertheless, the results shown above suggest that Asia is one of multiple probable places of origin.

Although numerous studies on genetic differentiation of *A*. *cantonensis* have been carried out recently [11, 14–18], only two of these were based on analysis of the sequences of cytochrome b (Cytb) as a means of evaluating the genetic differentiation of this parasite [17, 18]. The complete mitochondrion genome has been reported for *A*. *cantonensis* from the Chinese mainland (GenBank accession number GQ398121—complete mitochondrial genome) and from Thailand (GenBank accession number KT186242—complete mitochondrial genome), which therefore allowed us to study the phylogeography of this parasite based on variable mitochondrial gene sequences in different areas of China in the geographic range where it was initially reported. This species has not been previously investigated for its molecular phylogeography in China.

The objective of the current study is to reveal the pattern and processes of geographic distribution of *A*. *cantonensis* in China. Our analysis focused on the Cytb gene of *A*. *cantonensis* from *Achatina fulica* mainly collected from southern China (including sampling locations in Taiwan) and the surrounding region, with some samples from Laos (Vientiane) close to northern Thailand included.

## Materials and methods

### Ethics statement

All the samples for the present study are isolated from snails which did not concern with ethnic issue. All the experimental manipulation accorded with animal safety and ethnic rule issued by the School of Life Sciences, Sun Yat-sen University.

### Sample collection and DNA extraction

**Fig 1:**
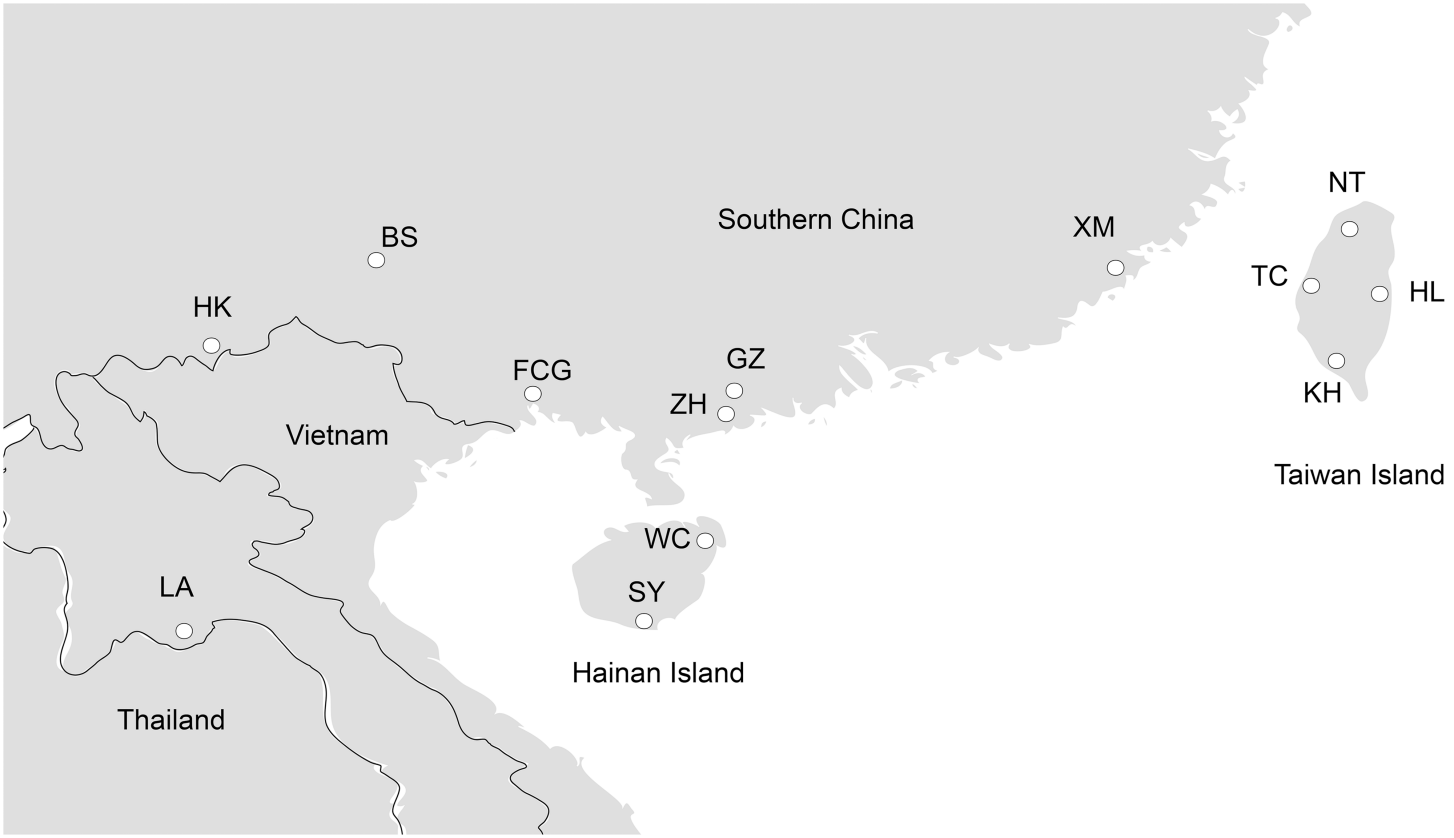
Map showing sample localities for *A*. *cantonensis*. South China mainland (GZ, Guangzhou; ZH, Zhuhai; XM Xiamen; FCG, Fangchenggang; BS, Baise; HK, Hekou), Hainan Island (SY, Sanya; WC, Wenchang), Taiwan Island (NT, New Taipei; TC, Taichung; KH, Kaohsiung; HL, Hualien) and Laos (adapted from Wikimedia Commons).

Specimens of *Achatina fulica*, identified by morphological characteristics, were captured from 13 different locations in southern China and Laos. These samples were collected in Guangzhou (GZ, 23°7', 113°15'E), Zhuhai (ZH, 22°16'N, 113°34'E), Xiamen (XM, 24°29'N, 118°05'E), Fangchenggang (FCG, 21°41'N, 108°21'E), Baise (BS, 23°54'N, 106°37'E), Hekou (HK, 24°15'N, 106°17'E), Wenchang (WC, 30°33'N, 114°19'E; Hainan Island), Senya (SY, 18°15'N, 109°31'E; Hainan Island), New Taipei (NT, 25°03'N, 121°31'E), Taichung (TC, 24°09'N, 120°41'E), Kaohsiung (KH, 22°37'N, 120°17'E), Hualien (HL, 23°58'N, 120°36'E) and Laos (LA, Vientiane, 17°58'N, 102°37'E), as presented in Fig 1 for details. The snails captured were transported to field laboratory facilities for parasite examination in a ventilated, humid container. The sampling was conducted either between 20:00 and 24:00 or 6:00 and 8:00 between July 2013 and November 2015. To examine the third stage larvae (L_3_), snails were individually dissected after surface cleaning. The pulmonary membrane and part of mesenterium were obtained from each snail. After treatment in a bottle with 50 ml digestive solution (2g/L 1:3000 pepsin, 0.7% HCl) [19] for 15 min, the digested samples were stirred into pieces using a blender. Individual worms were obtained under microscopy and preserved in 70% ethanol for subsequent DNA extraction.

Genomic DNA was extracted from individual worms, strictly following the protocol provided by manufacturer (TIANamp Marine Animals DNA Kit). The final product extracted from each individual worm was preserved in 50 μl TE buffer solution (10 mM Tris-HCl, 1 mM EDTA) and stored at below -20°C.

### PCR amplification and sequencing

The DNA product extracted from each nematode was used as the template to amplify the complete mitochondrial cytochrome b (Cytb). Amplification was conducted by nested PCR reaction in a volume of 25 μL containing 2.5 μL of 10 × Ex Taq buffer, 1.5 mM of MgCl_2_, 0.2 μM of each dNTP, 1U of Ex Taq polymerase (TaKaRa, Japan), 0.2 μL of extraction product for the primary amplification, and 0.2 μl of preliminary product for the subsequent nested reaction. Each reaction was carried out in a Biometra thermal cycler (TC-96/G, BIO-DL) using the following procedure: 3 min at 94°C for denaturation, followed by 35 cycles of 30 s at 94°C, 30 s at 50–53°C for annealing, 90 s at 72°C, and a final extension at 72°C for 3 min. The primers used for amplification are listed in supplementary S1 Table. Final products were analyzed by electrophoresis on 1.5% agarose gel and visualized under UV light staining with ethidium bromide.

PCR products were purified with the UNIQ-10 Spi Column PCR Product Purification Kit (Sangon, China) and subjected to automated DNA sequencing (BGI, China) with the same primers used for nested amplification.

## Data analyses

### Genetic diversity and phylogenetic relationships

Nucleotide sequences were compiled and aligned in MEGA 6.0, followed by visual inspection. Nucleotide sequences were then translated into acid sequences with the invertebrate mitochondrial code to ensure that no unclear pseudogenes were amplified.

Dnasp 5.0 [20] and Arlequin 3.5 [21] were used to evaluate molecular diversity through the number of haplotypes (*H*), polymorphic sites (*S*), haplotype diversity (*h*), nucleotide diversity *(p*) and the average numbers of pairwise nucleotide differences (*k*). Pairwise and overall distances among haplotype sequences were calculated in MEGA 6.0 [22].

The best-fit substitution model for Cytb gene data and the gamma distribution parameter for the rate of heterogeneity among sites were determined using Modeltest 3.07 [23] based on the Hierarchical Likelihood Ratio Tests (hLRTs). The TrN model [24] of evolution with the gamma shape parameter (TrN + G) was selected for the subsequent analysis of molecular variances (AMOVA) and phylogenetic analysis.

Neighbor-joining (NJ) trees were constructed using MEGA 6.0 with *Angiostrongylus costaricensis* (Genbank: GQ398122) as outgroup. The genetic distances were estimated under Tamura and Nei model of substitution suggested by Modeltest. Bootstrapping was conducted with 1,000 replicates [25] as the assessment support for the NJ tree. The calculated best-fit parameters were adopted to reconstruct the Bayesian tree in MrBayes 3.2.1 [26]. Four Markov Chain Monte Carlo (MCMC) were run for 100,000,000 generations sampled every 100 generations with a burnin value of 250,000. Maximum likelihood phylogenetic tree was generated using PhyML 3.1 [27] with BIONJ methods and bootstrap analysis (1,000 replicates). The haplotype network was constructed with Popart 1.7 [28] using the median joining network (MJN) approach [29].

### Population structure

The AMOVA was undertaken to describe the population structure, which was implemented in Arlequin 3.5 by F-statistics at three subdivided geographical hierarchical levels: the proportions of variations among regions (FCT′), among populations within region (FSC′) and within populations (FST′) with a 5,000-times permutation to assess the significance of the covariance components associated with the different possible levels of genetic structure. Both the calculation of fixation index (FST) with 10,000 permutations and statistical significance were used to evaluate the genetic differentiation between pairwise populations. The genetic distances between haplotypes was revised under the Tamura and Nei model of nucleotide substitution with a gamma shape parameter (G = 0.3017) suggested by Modeltest. A comparison between the observed distribution frequency and the expectations under panmixia [30] was conducted as the exact test of the differentiation of haplotypes among populations, which is aimed to test the null hypothesis of population panmixia. Probabilities were estimated by permutation analyses using 10,000 randomly permuted r (populations) × k (different haplotypes) contingency tables of haplotype frequencies. All statistics described above were performed in Arlequin 3.5.

### Demographic analysis

Two different methods were adopted to the historical demographic analysis. First, the frequency distribution of pairwise differences among all haplotypes (mismatch distribution) was tested under the sudden expansion model of Rogers [31]. Deviations from the estimated demographic model were evaluated by the tests of Harpending’s raggedness index [32] and the sum of squared differences (*SSD*) with a parametric bootstrapping approach using 10,000 replicates. The mismatch distribution of samples drawn from populations at demographic equilibrium is usually multimodal while that for samples from populations with recent demographic and distributional expansions is usually unimodal [33]. Given that mismatch distributions could be very conservative occasionally [34], both Tajima’s *D* [35] and Fu’s *F*s [36] tests based on neutral hypothesis were carried out under coalescent simulation algorithm in Arlequin 3.5. Tajima’s *D* test compares two estimators of the mutation parameter *θ*: Watterson’s estimator *θ*s and Tajima’s estimator *θ*π; significant *D* values are typically generated as the result of factors such as population expansion, bottlenecks and selection. In Fu’s *F*s test, the number of haplotypes observed is contrasted with that expected in a random sample under the assumption of an infinite-sites model without recombination. Additionally, *F*s is sensitive to demographic expansion, which generally results in a negative *F*s value.

## Results

### Genetic diversity

**Fig 2:**
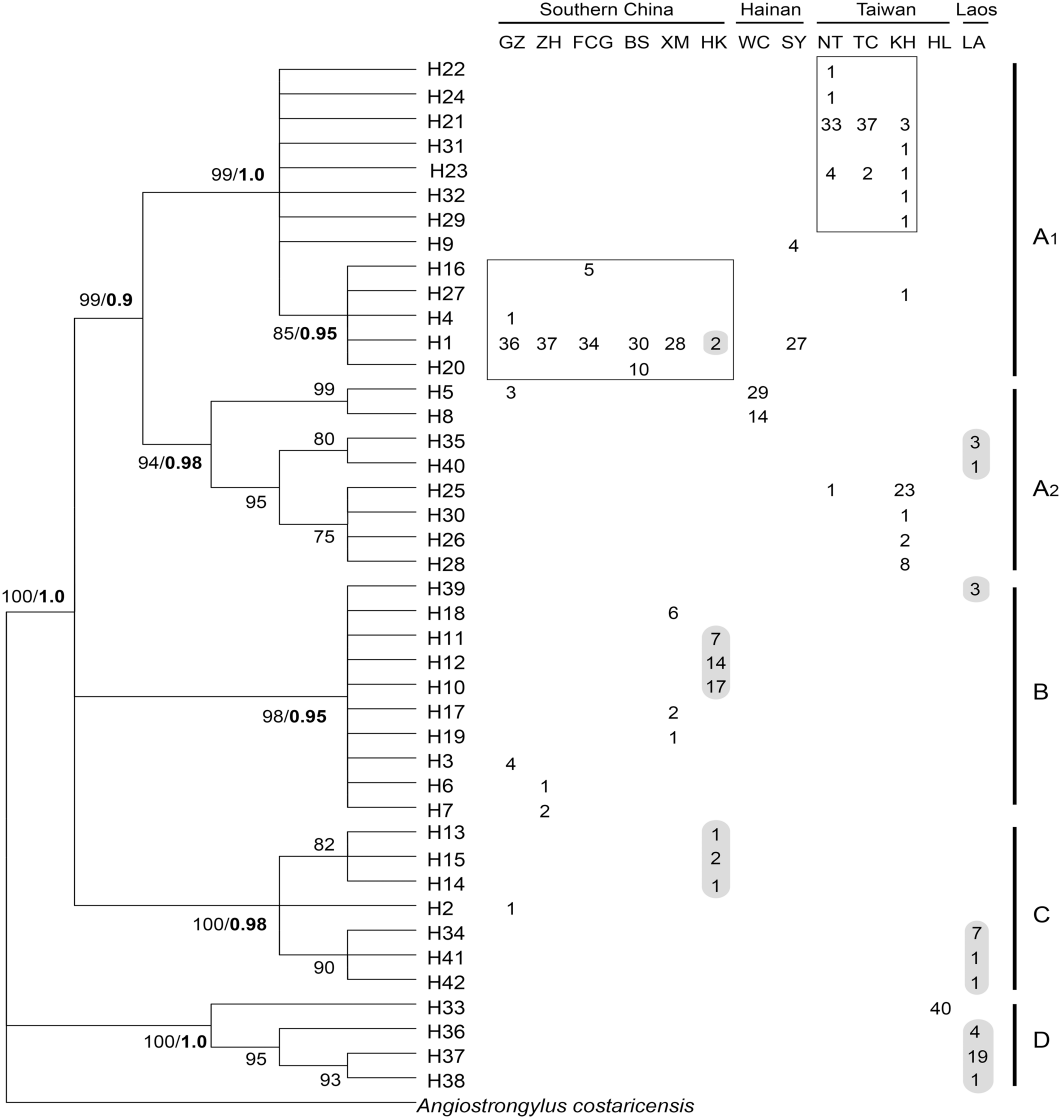
Neighbour joining tree for Cytb haplotypes of *A*. *cantonensis*. Bootstrap supports of >75% (left) and Bayesian posterior probabilities (right in bold) for main clades were labelled at nodes. The haplotype composition at each locality was presented by frequencies distribution on the right of NJ tree. A_1_, A_2_, B, C and D were 5 clades supported by high bootstrap values. Numbers in squares represented individuals from south mainland China and Taiwan while the numbers highlighted in gray were of Hekou and Laos, indicating the recognized geographic association of haplotypes.

Complete Cytb gene sequences of 1110 bp were obtained from 520 individuals of *A*. *cantonensis* in samples representing 13 populations. Among these sequences, a total of 42 haplotypes were identified with 229 polymorphic sites including 192 transitions, 44 transversions and 8 indels. Except for 5 haplotypes (H1, H5, H21, H23 and H25) shared by multiple localities, all others are private to their specific populations. H1 and H5 appear in southern China and Hainan Island, while the distributions of H21, H23 and H25 are restricted to Taiwan Island. H1 was the most prevalent haplotype (194 of 520) and occupied the widest range of localities (all sites excluding Taiwan and Laos). The H21 haplotype was the most commonly detected haplotype in Taiwan Island (Fig 2).

Genetic diversity indices of all regions are presented in Table 1 with an overall haplotype diversity (*h*) of 0.8253±0.0138 and nucleotide diversity (*π*) of 0.087096±0.041454, exhibiting a high level of haplotype diversity but low nucleotide diversity. Among regions, Laos and Hekou exhibited the highest haplotype diversities, 0.7385±0.0614 and 0.7357±0.0395 respectively, and the highest nucleotide diversities of 0.154297±0.074972 were also detected for samples from Laos. Samples from Xiamen and Hekou had considerably high values of nucleotide diversities, 0.029379±0.014567 and 0.022392±0.011127 respectively.

**Table 1:** Sampling localities, grouped regions and descriptive statistics of genetic diversity of *A*. *cantonensis*.

Region and site	Abbreviation	*N*	*H*	*S*	*h*	*π*	*k*
Gaungzhou	GZ	45	5	109	0.3545±0.0878	0.021615±0.010746	24.035787±10.762781
Zhuhai	ZH	40	3	63	0.1449±0.0737	0.011537±0.005899	12.805818±5.893904
Xiamen	XM	37	4	62	0.4084±0.0906	0.029379±0.014567	32.610748±14.549809
Fangchenggang	FCG	39	2	2	0.2294±0.0125	0.000426±0.000421	0.473296±0.420797
Baise	BS	40	2	1	0.3846±0.0698	0.000350±0.000373	0.388091±0.372518
Wenchang	WC	43	2	1	0.4496±0.0520	0.000409±0.000409	0.453677±0.409132
Sanya	SY	31	2	10	0.2323±0.0899	0.002283±0.001404	2.534559±1.400723
New Taipei	NT	40	5	37	0.3154±0.0914	0.002101±0.001303	2.332536±1.301627
Taichung	TC	39	2	1	0.0999±0.0635	0.000090±0.000174	0.100195±0.174319
Kaohsiung	KH	42	10	41	0.6690±0.0709	0.012488±0.006351	13.861821±6.347930
Hua Lien	HL	40	1	0	0.0000±0.0000	0.000000±0.000000	0.0000000±0.000000
Hekou	HK	44	7	95	0.7357±0.0395	0.022392±0.011127	24.855371±11.123840
Laos	LA	40	9	177	0.7385±0.0614	0.154297±0.074972	171.424310±74.979997
Total samples	Total	520	42	229	0.8253±0.0138	0.087096±0.041454	96.850705±41.681545

*N*, sample size; *H*, number of haplotypes; *S*, number of segregating sites; *h*, haplotype diversity (±S.D.); *π*, nucleotide diversity (±S.D.); *k*, mean pairwise difference (±S.D.).

### Phylogenetic analysis

**Fig 3:**
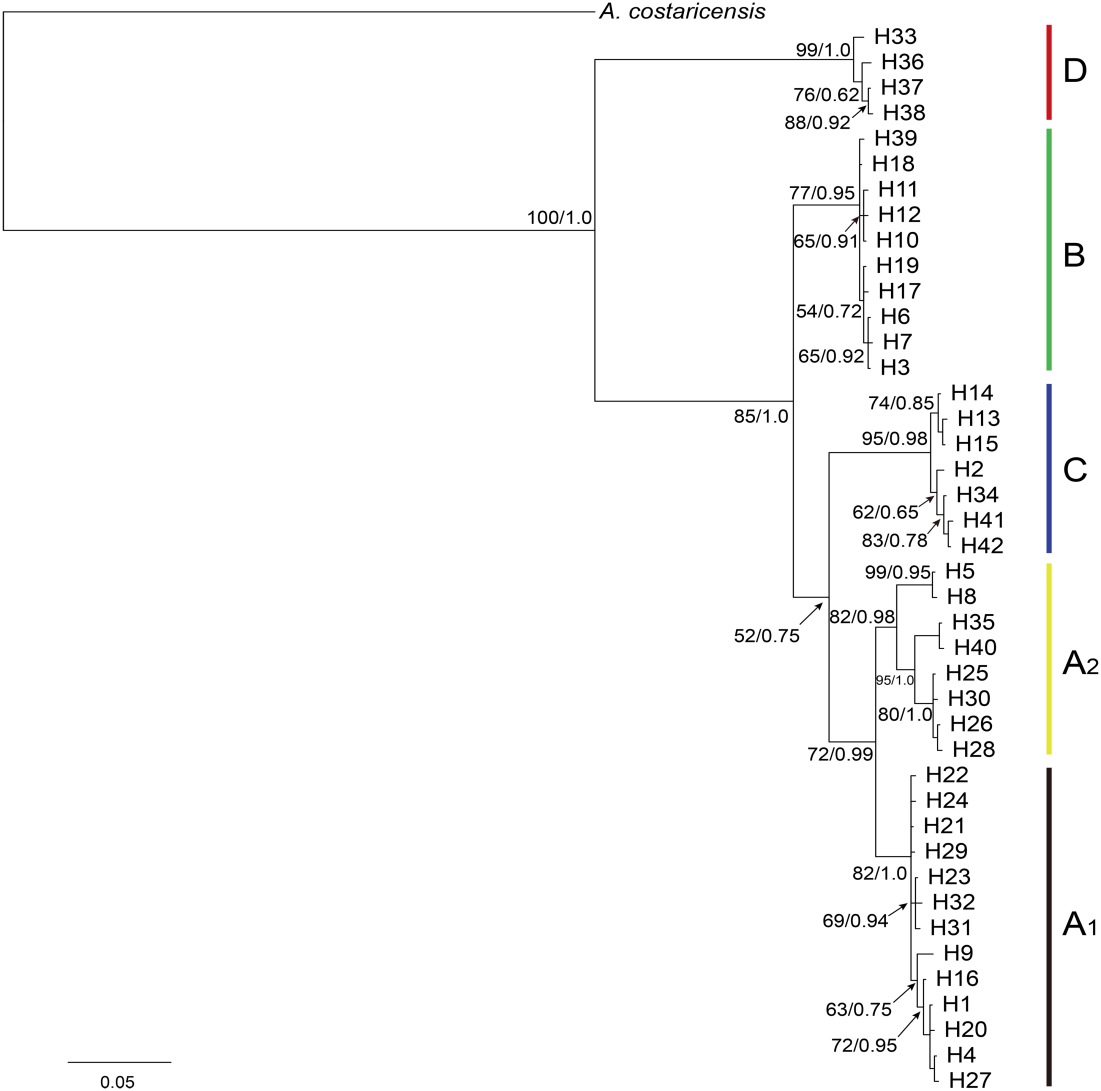
Bayesian tree for Cytb haplotypes of *A*. *cantonensis*. The posterior probabilities of Bayesian on the right and bootstrap values of maximum-likelihood with 1,000 replicates on the left (ML tree displays the same topology, S1 Fig) are shown at nodes as support values. Topology structure revealed here are similar to Fig 2.

Tamura and Nei with the gamma shape parameter (TrN + G, G = 0.301) was selected as the best-fit substitution model suggested by Modeltest to reconstruct the phylogenetic tree for Cytb haplotypes data (Fig 2). Haplotypes from 13 regions were scattered in 4 distinct clades with high support (bootstrap supports >75%): Clade A comprises haplotypes from all localities, whereas Clade B includes samples from GZ, ZH, XM, HK and LA; Clade C includes samples from GZ, HK and LA; while Clade D only consists of samples from HL and LA. Clade A was further divided into two subclades, A_1_ consisting of the majority of haplotypes from southern China with few from two islands (Taiwan and Hainan) and A_2_ including samples from GZ, WC, KH and LA. In spite of the farraginous composition in clade A, the majority of haplotypes from southern China were clustered with samples from Hainan Island and accordingly separated from haplotypes from Taiwan Island (squares displayed in Fig 2, NJ bootstrap support / Bayesian posterior probabilities as 85% **/** 0.95), implying a genetic divergence of haplotypes between Taiwan Island and southern mainland China. In contrast with Clade A, no significant connection between haplotype composition and sampling localities was found in the other 3 clades. Nonetheless, the distribution of haplotypes among clades was tendentious and meaningful: haplotypes form HK interspersed among Clade A_1_, B and C; GZ interspersed among Clade A, B, and C; LA interspersed among Clade A_2_, B, C and D. Phylogeny of haplotypes was more distinctly and remarkably revealed in Bayesian analysis (Fig 3). The tree topology with branch length showed the same pattern consists of 5 clades with high posterior probabilities and bootstrap support. Noteworthy, neighbour-joining as well as Bayesian analysis revealed the strongly supported monophyletic Clade D.

**Fig 4:**
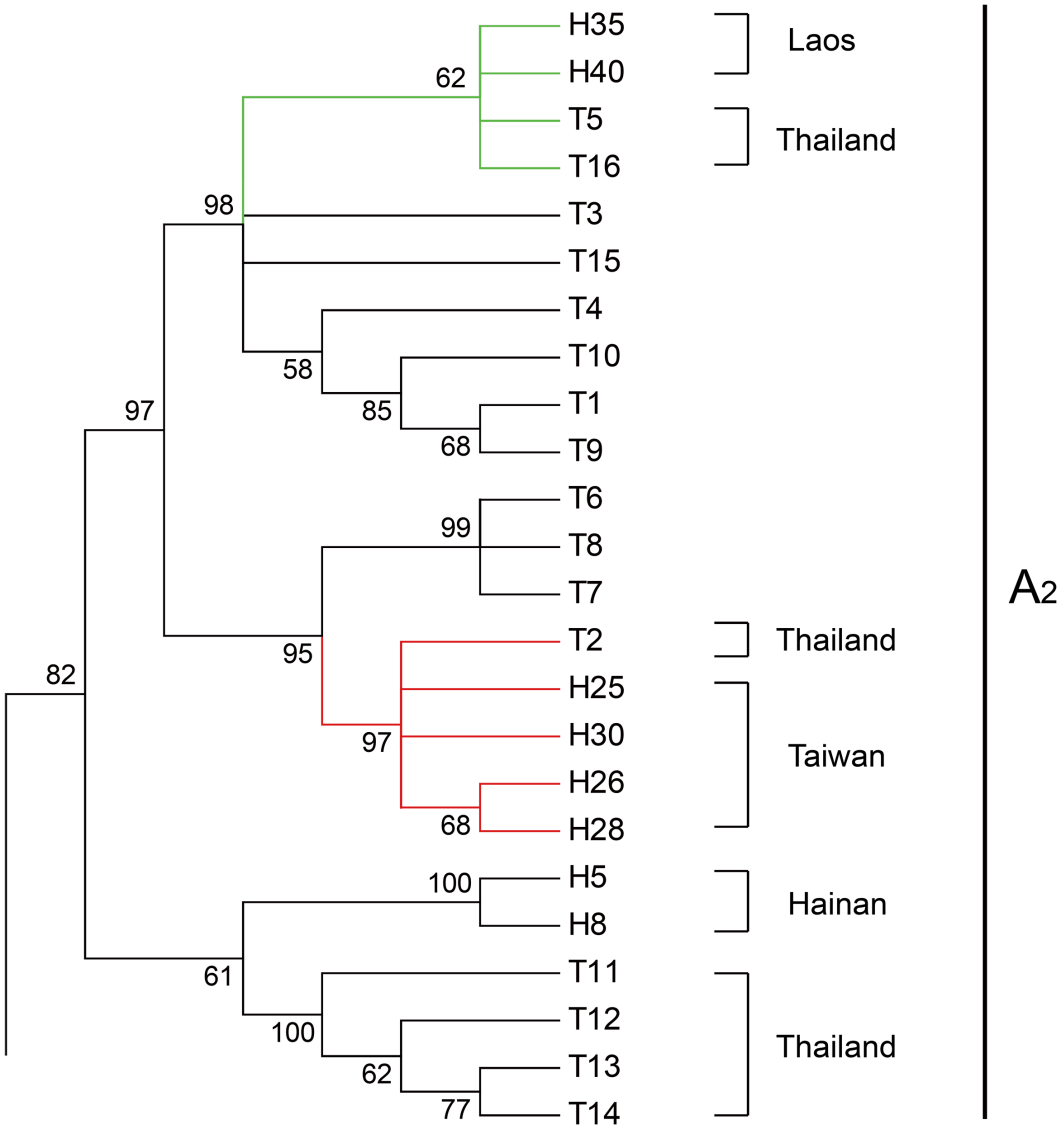
Variable part of neighbor-joining tree for partial Cytb sequence with haplotypes from Thailand. Bootstrap supports of >50% are shown at nodes. Comprising haplotypes from Laos, Taiwan, Hainan and Thailand, a clade presented here corresponds to Clade A_2_ in Fig 2. T1-T16 represent haplotypes from Thailand. Among them, T5 and T16 were included in the cluster of Laos, T2 was in that of Taiwan, which were respectively highlighted in green and red, while T11-T14 were in the cluster of Hainan Island, China.

The NJ tree for partial Cytb sequences with additional haplotypes from Thailand obtained from GenBank under accession number KP721442—KP721453 was constructed using the same parameters (TrN + G, G = 0.301). All the haplotypes from Thailand were interfused into Clade A_2_, forming a distinctive clade with haplotypes from Laos, Hainan and Taiwan regions, as shown in Fig 4. This strongly suggests the close relationship of haplotypes from Taiwan and Thailand.

**Fig 5:**
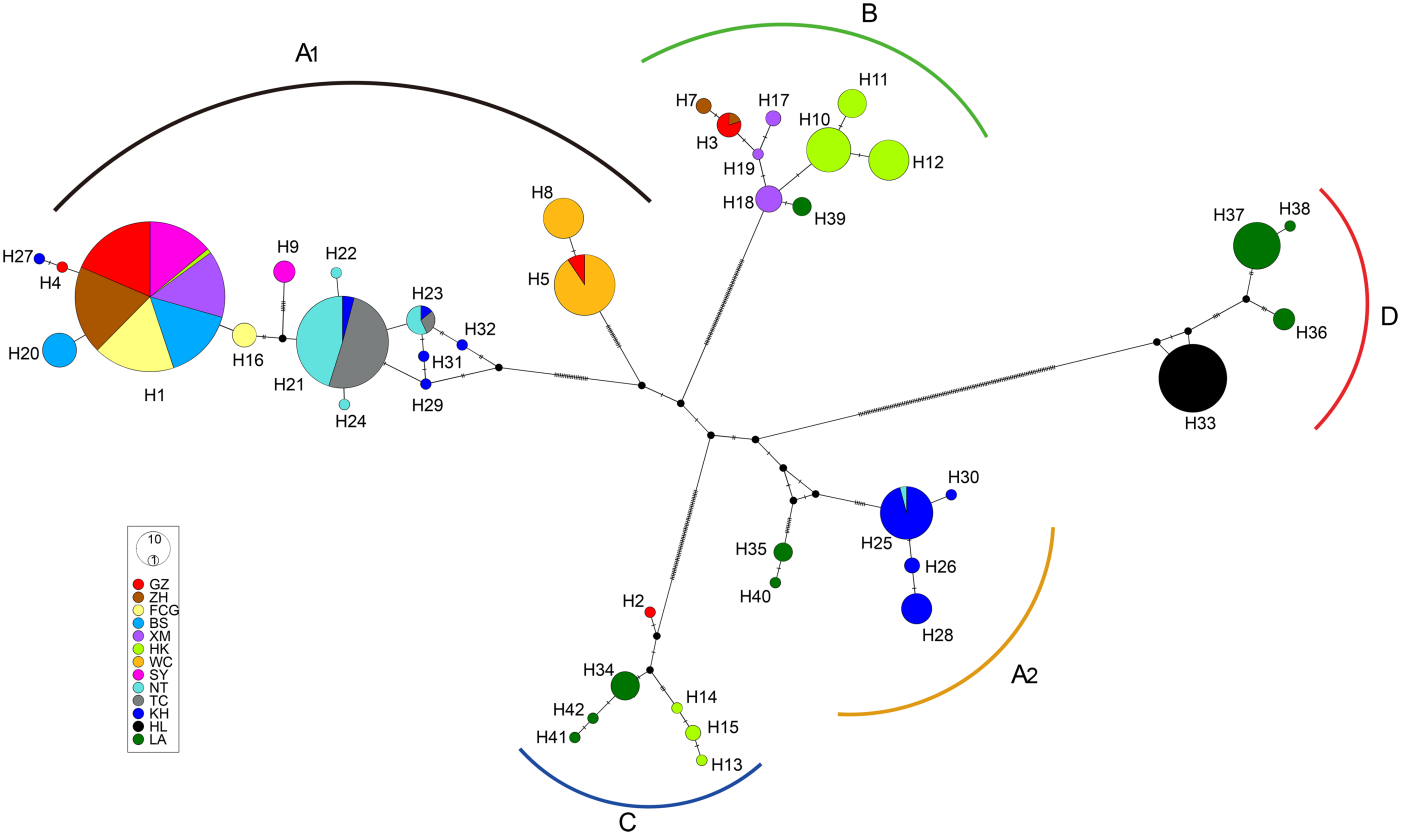
Median-joining network for Cytb haplotypes of *A*. *cantonensis*. The size of the circles represents the haplotype prevalence and the colors stand for the populations to which they belong, black dots the missing intermediate haplotypes within clades and small transversal lines the mutational steps. Five clades consisting of specific haplotypes correspond to those in the phylogenetic trees.

The network analysis similarly revealed that the haplotypes nested into 5 clusters (Fig 5) corresponding to the clades obtained from phylogenetic tree analysis. Clade D was confirmed to be significantly distinguished with other clades by the branch length and mutational steps between them. Additionally, a cluster corresponding to Clade A_2_ appeared to be closer to Clade D than that to Clade A_1_ as displayed in the phylogenetic tree.

### Population structure

Genetic differentiation among populations (*Fst*) was summarized in Table 2. All except five pairwise comparisons of *Fst* revealed significant differences (*P*<0.05), implying the existence of significant population structure across the range investigated. These results were further confirmed by hierarchical AMOVA tests which attributed nearly 50% of the genetic variation to the variabilities among populations within groups (Table 3). Besides, the between-group differences for the two groupings accounted for less variability than those between-populations in groups, which was similarly revealed by exact test. Hence, the null hypothesis was not applicable, or in other words, these results taken together suggest that populations of *A*. *cantonensis* in southern China are not panmictic.

**Table 2:** Pairwise *F*_*ST*_ (below diagonal) and *P* values for exact test of population differentiation (above diagonal) among populations.

	HK	LA	GZ	ZH	FCG	BS	XM	WC	SY	NT	TC	KH	HL
HK		0.01689	0.00254	0.00015	0.00060	0.00000	0.00000	0.01171	0.00000	0.00000	0.00000	0.00000	0.00000
LA	0.56336*		0.02241	0.00061	0.00098	0.00000	0.00000	0.02650	0.00000	0.00000	0.00000	0.00000	0.00000
GZ	0.69205*	0.58138*		0.00006	0.00034	0.00000	0.00000	0.00567	0.00000	0.00000	0.00000	0.00000	0.00000
ZH	0.76069*	0.59649*	-0.00238		0.00000	0.00000	0.00000	0.00044	0.00000	0.00000	0.00000	0.00000	0.00000
FCG	0.84223*	0.62095*	0.07318*	0.04444		0.00000	0.00000	0.00300	0.00000	0.00000	0.00000	0.00000	0.00000
BS	0.84606*	0.62703*	0.08774*	0.06050*	0.16673*		0.00000	0.00000	0.00000	0.00000	0.00000	0.00000	0.00000
XM	0.57841*	0.53919*	0.03208	0.07573	0.21823*	0.23187*		0.00000	0.00000	0.00000	0.00000	0.00000	0.00000
WC	0.86166*	0.62492*	0.75349*	0.87579*	0.99035*	0.99131*	0.73310*		0.00000	0.00000	0.00000	0.00000	0.00000
SY	0.81994*	0.59096*	0.06093*	0.04275	0.06815	0.13588*	0.18995*	0.97250*		0.33330	0.00000	0.00000	0.00000
NT	0.82872*	0.61381*	0.28736*	0.44748*	0.81159*	0.83124*	0.33717*	0.96742*	0.69797*		0.00000	0.00000	0.00000
TC	0.83920*	0.61505*	0.30678*	0.48533*	0.95493*	0.96446*	0.35435*	0.99317*	0.82632*	-0.00229		0.00000	0.00000
KH	0.78495*	0.59844*	0.58347*	0.69991*	0.81609*	0.82219*	0.56425*	0.78763*	0.78001*	0.75498*	0.78892*		0.00000
HL	0.95807*	0.32216*	0.95894*	0.97940*	0.99925*	0.99938*	0.94912*	0.99920*	0.99645*	0.99621*	0.99984*	0.97617*	

*, Indication of significant difference, *P* < 0.05

**Table 3:** Summary of hierarchical analysis of molecular variances for *A*. *cantonensis*.

	Source of variation	Variance components	Percentage of variation	F statistics	*P* value
Grouping A	Among groups	10.53388	34.03	FCT = 0.34029	0.16389
	Among population within groups	16.72340	54.02	FSC = 0.75582*	0.00000
	Within populations	3.69836	11.95	FST = 0.88053*	0.00000
Grouping B	Among groups	12.74203	36.10	FCT = 0.36096	0.16369
	Among population within groups	16.66807	47.22	FSC = 0.73890*	0.00000
	Within populations	5.88981	16.69	FST = 0.83315*	0.00000

Grouping A: HK with other isolations (GZ, ZH, FCG, BS, XM, WC, SY, NT, TC, KH, HL) as 2 groups.

Grouping B: LA with other isolations (GZ, ZH, FCG, BS, XM, WC, SY, NT, TC, KH, HL) as 2 groups.

*, Indication of significant difference, *P* < 0.05

### Historical demography

**Fig 6:**
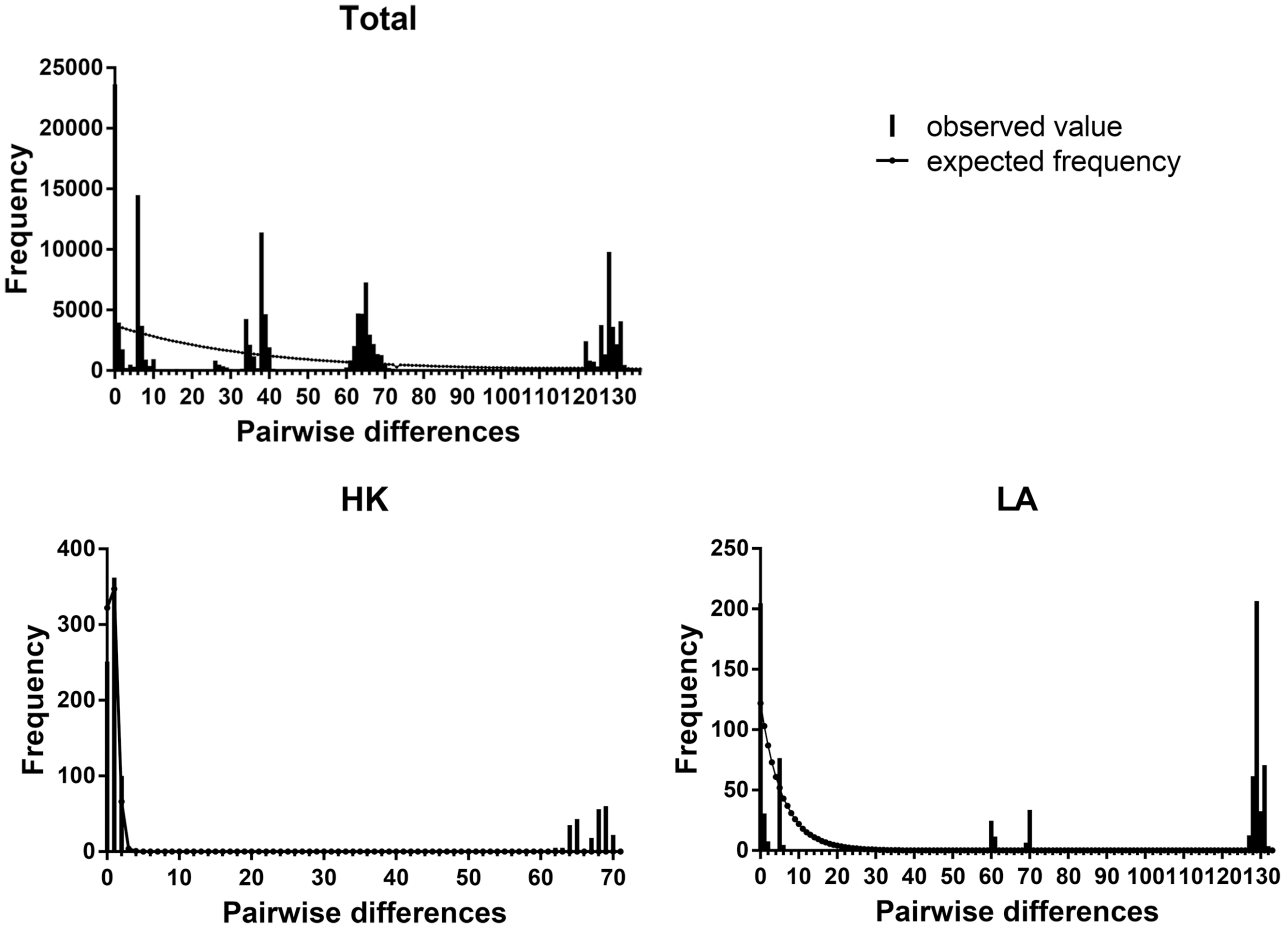
Mismatch distribution of Cytb haplotypes of *A*. *cantonensis* in three regions. The observed pairwise differences were shown as bars and the expected values under the sudden expansion model were in solid line.

Despite a lack of significance of the goodness of fit test (*HRI*, *P* > 0.05) of the distribution from that expected under the expansion model (Table 4), the mismatch distribution of pairwise differences among regions exhibited irregular multimodal patterns rather than the unimodal pattern generally produced by populations that have experienced demographic expansion (Fig 6). The results of neutrality tests were consistent with the observed mismatch analyses. Neither *F*s statistic of all regions nor Tajima’s *D* statistic of most regions revealed any significant difference (*P* > 0.05) from that under neutral assumption. These results lead us to conclude that populations of *A*. *cantonensis* in the range studied were inconsistent with stable demographic history, although no strong evidence emerged in support of episodes of expansion.

**Table 4:** Tajima’s *D*, Fu’s *F*_S_ statistics, corresponding *P* values and mismatch distribution parameter estimates for *A*. *cantonensis*.

	Tajima’s *D*		Fu’s *F*s		Goodness-of-fit tests			
	*D*	*P*	*Fs*	*P*	*SSD*	*P*	*HRI*	*P*
GZ	-1.12731	0.11700	24.38064	1.00000	0.07065	0.07500	0.43610	0.52400
ZH	-1.43256*	0.04810	18.09387	0.99980	0.02053	0.06400	0.74037	0.74200
FCG	-0.05710	0.43820	1.61846	0.71750	0.07893	0.07700	0.69906	0.26800
BS	0.90802	0.87300	1.29156	0.62600	0.00596	0.19500	0.20118	0.25300
XM	1.98791	0.98800	29.80143	1.00000	0.13130	0.05300	0.46359	0.42500
WC	1.32256	0.91940	1.63083	0.71310	0.01289	0.09600	0.21231	0.08300
SY	-0.22680	0.45550	7.01286	0.99180	0.07724*	0.02100	0.69732	0.63300
NT	-2.69931*	0.00000	1.72916	0.81260	0.00694	0.42200	0.24746	0.60500
TC	-0.83095	0.20090	-0.66622	0.11700	0.00006	0.26000	0.65040	0.78000
KH	0.75673	0.82380	6.90517	0.97400	0.53343*	0.00000	0.12162	0.99900
HL	0.00000	1.00000	0.00000	N.A.	0.00000	0.00000	0.00000	0.00000
HK	-0.86611	0.19890	17.87902	0.99980	0.03147*	0.04500	0.10855	0.10700
LA	2.50836	0.99840	0.00000	1.00000	0.14561	0.35900	0.17146*	0.04700
Mainland	0.77350	0.83410	26.80099	0.99230	0.31918*	0.00000	0.18295	0.99700
Taiwan	3.64168	0.99980	0.00000	1.00000	0.13867*	0.01200	0.18966	0.07800

Mainland: GZ, ZH, FCG, BS, XM, HK.

*SSD*, Sum of square differences; *HRI*, Harpending’s raggedness index

*, Indication of significant difference, *P* < 0.05

## Discussion

Because this parasitic species is associated with severe tropical diseases, extensive research has been conducted on *A*. *cantonensis*, particularly regarding clinical pathology, epidemiology and diagnosis [37–40], however, studies on its spread mechanism and genetic variation have obtained relatively little attention. Cytb nucleotide sequence has been adopted for the phylogenetic studies of *A*. *cantonensis* [17, 18]. This gene sequence has also been accepted as good marker to reveal phylogeographical patterns [41, 42].

From 520 Cytb sequences (1110 bp) of *A*. *cantonensis* sampled from 13 geographic regions, a total of 42 haplotypes were identified. The intraspecific diversity of haplotypes among all regions ranged from 0.1 to 14.0% in p-distance with an overall mean value of 6% (Tamura and Nei distance). High level values were generated between Clade D (H33, H36, H37, H38) and other clades (haplotypes) as 12.8 to 14.0%, while those of haplotypes within other three clades only ranged from 0.1 to 6.8% (S2 Table). This was corroborated by the phylogenetic tree in which these four distinct haplotypes (H33, H36, H37, H38) were grouped into a monophyletic Clade D. The complete mitochondrial genomic sequence of *A*. *cantonensis* collected from Thailand also demonstrated that *A*. *cantonensis* from Thailand is distinctly isolated from the population in China with p-distance of 11.6% [43]. Newly published results have revealed the existence of two dramatically divergent lineages of *A*. *cantonensis* in Thailand based on mitochondrial and nuclear sequences data with an average of 11% p-distance, which is in agreement with our results, since the sample site in Laos is adjacent to Thailand [44]. Such remarkable intraspecific divergence is worthy of further attention.

Numerous species of rodents serve as the definitive hosts of *A*. *cantonensis*, and these organisms are capable of highly promoting the spread and intraspecific transfer of this parasite. The genetic structure of populations from different regions was still different from those under random mating in some way. By contrast, owing to the high dispersal potential and lack of geographic barrier in the marine environment, marine organisms normally do not display evidence of genetic differentiation throughout wider geographic range and consequently their parasites typically exhibit a similar genetic pattern [45]. The phylogeography of *Pseudokuhnia minor*, a species of monogenean on the host of chub mackerel *Scomber japonicus*, exhibited no significant genetic structure along the coast of China, implying panmixia within the range of the population [46]. As the parasitic organisms depend on their hosts, the biological characteristics of their hosts can profoundly impact their population genetic structure, in addition to their own dispersal ability [47]. Although *A*. *cantonensis* has many definitive and intermediate hosts, these hosts have limitations in traversing habitat barriers, such as water for *Achatina fulica* and low temperature on the high mountains for both *Achatina fulica* and *Pomacea canaliculata*. Inevitably, the definitive host rodents have complex impacts on the population genetic structure of *A*. *cantonensis* because of their wide adaptation in terrestrial habitats, which is in need of further study.

Notably, haplotypes from Hualien (HL) and Laos (LA) generated Clade D which substantially diverges from other clades, implying the population from Hualien is a unique lineage in Taiwan. The exact test and low level of genetic diversity of population from Hualien also revealed the absence of gene flow between Hualien population and populations in other regions. Another survey on the genetic diversity of *A*. *cantonensis* in Taiwan based on partial COI sequence data also revealed the existence of a distinct strain of *A*. *cantonensis* in Hualien, which is distinguished from strains in other regions in Taiwan by not only the genetic distance, but also discrepancies in infectivity and pathogenicity [48]. Hence, we inferred that the separation between western and eastern Taiwan by the central mountain range presents a substantial geographical barrier; this, combined with the limited propagative capability of hosts simultaneously shaped the unique distribution pattern of *A*. *cantonensis* in Taiwan.

The unique lineage of the Hualien strain showed a closer relationship with lineages from Laos (p-distance = 6% - 7%) indicating that they might share a common origin, despite the thousands-miles separation by the Pacific Ocean between these two locations. This unusual pattern may be attributed to the worldwide dispersal of the hosts of this parasite as influenced by human activities. With regard to the spread of the widely introduced and invasive land snail species around the world, *Achatina fulica*, it was proposed that numerous impolitic introduction and activities of the Japanese army during World War II figured importantly in establishing its range [49, 50]. Phylogeographic study on the introduced *Rattus rattus* in the western Indian Ocean islands also revealed effects of human-mediated colonization [51]. The inference that *A*. *cantonensis* has established global-scale dispersal with various organisms/hosts or vectors that are largely influenced by human transportation has gained wide acceptance [10, 11, 17]. Besides the situation in Hualien, the association between *A*. *cantonensis* populations along the west coast of Taiwan Island with those in Thailand are also supported by the reconstructed phylogeographic tree (Figs 2 and 4). In conclusion, it could be inferred that Southeast Asia is a likely origin of *A*. *cantonensis* in Taiwan, but with variable and independent introductions.

In the study of invasion biology, we generally consider introduced populations as having low genetic variability [52, 53]. This could be observed in the survey on population genetics of invasive American bullfrog *Lithobates catesbeianus* that genetic diversity was greatly reduced in colonizing populations due to demographic bottlenecks [54]. Hekou and Laos are two sampling locations where the *A*. *cantonensis* populations have significant high levels of genetic diversity (Table 1). It can generally be deduced that *A*. *cantonensis* has higher genetic diversity in its origin area than the diversity seen among more recently established populations with regard to the fact that its host *Achatina fulica* is a notorious invasive snail species. Moreover, these two locations were highlighted since phylogeny reveals multiple relationships between populations from these two sites and other sites as displayed in Figs 2 and 3. These results therefore support our inference that Hekou and Laos are the most likely origin of *A*. *cantonensis* in our study region, and further speculation that Southeast Asia might be a potential site of origin in Asia. Although the *A*. *cantonensis* population in KH (Taiwan) was clustered together with those from Thailand and Laos (Fig 4), the populations along west coast of Taiwan have close association with those in southern China (Clade A_1_), the latter were also closely linked to *A*. *cantonensis* in Hekou (Clade B). Hence, it could be proposed that Hekou is more likely the site of the origin of *A*. *cantonensis* in southern mainland China. It is still unknown how the population of *A*. *cantonensis* in Hekou may have been genetically connected to populations in Laos or Thailand, an understanding that may require further insight into the potential interaction of *A*. *cantonensis* populations between these locations.

With regard to the notable high genetic diversities of *A*. *cantonensis* populations in Guangzhou (GZ) and Xiamen (XM), it is likely that these port cities have a high probability of accepting the *A*. *cantonensis* carrying organisms, as it has been observed that epidemics frequently break out initially in port areas [55, 56]. It is also potentially relevant that multiple introductions of *A*. *cantonensis* from diverse regions have increased the genetic diversity in these port cities, at least in part as a consequence of human activities that influence the distribution of *A*. *cantonensis*.

Many relevant studies based on mtDNA sequence could reveal patterns of demographic history or association with known historic events [57–61], however, the present study based on a single mtDNA locus can only partially reveal the demographic history of this nematode, since the intricate life cycle of this parasite involves many species of snails as intermediate hosts and numerous rodents as definitive hosts. Besides, the limited documented records about its ecology and distribution also restrict our capacity to retrace its origin and spread. For better understanding of its phylogeography, further surveys concentrating on the meta-population of *A*. *cantonensis* in different species of hosts and based on more molecular markers will be required.

## Conclusion

The remarkable genetic differentiation between isolations indicated a low gene flow among the populations of *A*. *cantonensis* in different areas of Southern China. The dispersal via human activities across ocean, coupled with the natural spread of its hosts might have led to the establishment of several separated lineages in Taiwan Island. Additionally, no significant indication of demographic expansion was detected in the scope of survey, although significant genetic variation of *A*. *cantonensis* was found between southern China and Southeast Asia. The structured pattern of phylogenetic lineages has no precise correspondence to geographic distribution, underscoring the complicated nature of the task of tracing the population dynamics of this species. More samples from Southeast Asia and further survey of its host organisms together could provide more detailed evidence, leading to a higher degree of resolution in our understanding of the phylogeography of this parasite.

## Supporting information

S1 Table Primers for PCR amplification of Cytb gene. (DOCX)

S2 Table Genetic distance between haplotypes generated by MEGA 6.0. (XLS)

S1 Fig Maximum-likelihood tree based on haplotypes of Cytb data. (TIF)
