# Supplementary material for: Phylogeography of Angiostrongylus cantonensis (Nematoda: Angiostrongylidae) in southern China and some surrounding areas
Source: PLoS Negl Trop Dis. 2017 Aug 21;11(8):e0005776. doi: 10.1371/journal.pntd.0005776 (PMC5578690; doi:10.1371/journal.pntd.0005776)
Supplement: S1 Table — (DOCX) [file pntd.0005776.s001.docx]

**S1 Table.** Primers for PCR amplication of Cytb gene

|  | GQ398121 | |  | KT186242 | |
| --- | --- | --- | --- | --- | --- |
| primary | CBF | 5' GATTACGGTTCAGAAAGGTG 3' |  | CF-1 | 5' AAAGAAATATCTTTACCTCATAA 3' |
|  | CBR | 5' GGCAAATACACCCCAAACTT 3' |  | CR-1 | 5' TATTTATGTTATCTTGATAAGGTAG 3' |
| nested | CBF2 | 5' TTGGTGTAAAGGGGGGTT 3' |  | CF-2 | 5' GGATCAGAAAGGTGAACAT 3' |
|  | CBR2 | 5' GATACAAAACACAAATTATTCCT 3' |  | CR-2 | 5' AGAACTAGTAACATCTAAAGTCAT 3' |
