# Supplementary figures and images for: Phylogeography of Angiostrongylus cantonensis (Nematoda: Angiostrongylidae) in southern China and some surrounding areas
Source: PLoS Negl Trop Dis. 2017 Aug 21;11(8):e0005776. doi: 10.1371/journal.pntd.0005776 (PMC5578690; doi:10.1371/journal.pntd.0005776)

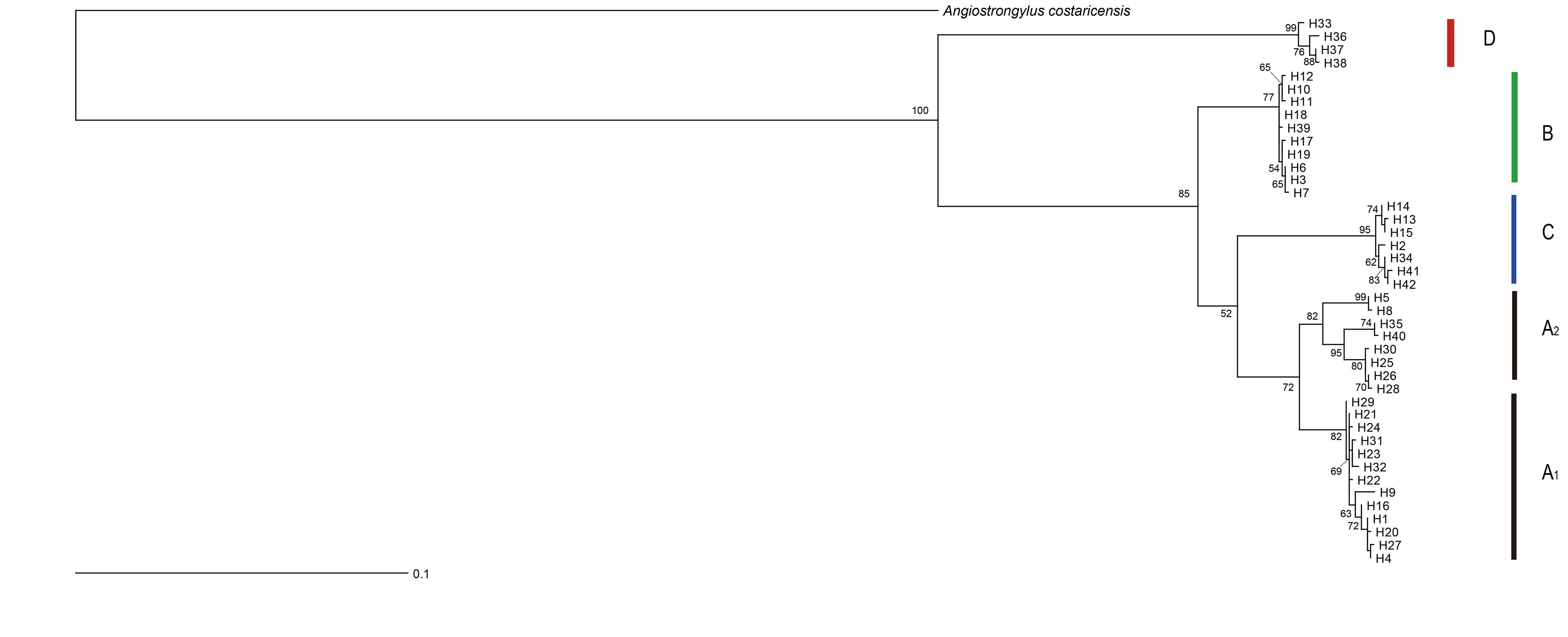

Supplement: S1 Fig — (TIF) [file pntd.0005776.s003.tif]
